# Histone 2-Hydroxyisobutyryltransferase Encoded by *Afngg1* Is Involved in Pathogenicity and Aflatoxin Biosynthesis in *Aspergillus flavus*

**DOI:** 10.3390/toxins15010007

**Published:** 2022-12-21

**Authors:** Jing Wang, Liuke Liang, Shan Wei, Shuaibing Zhang, Yuansen Hu, Yangyong Lv

**Affiliations:** 1 College of Biological Engineering, Henan University of Technology, Zhengzhou 450001, China; 2 Henan Provincial Key Laboratory of Biological Processing and Nutritional Function of Wheat, Zhengzhou 450001, China

**Keywords:** *Aspergillus flavus*, aflatoxin, pathogenicity, 2-hydroxyisobutyryltransferase, Afngg1

## Abstract

Aflatoxin, a carcinogenic secondary metabolite produced by *Aspergillus flavus*, is a significant threat to human health and agricultural production. Histone 2-hydroxyisobutyrylation is a novel post-translational modification that regulates various biological processes, including secondary metabolism. In this study, we identified the novel histone 2-hydroxyisobutyryltransferase Afngg1 in *A. flavus*, and explored its role in cell growth, development and aflatoxin biosynthesis. *Afngg1* gene deletion markedly decreased lysine 2-hydroxyisobutyrylation modification of histones H4K5 and H4K8 compared with the control strain. Additionally, *Afngg1* deletion inhibited mycelial growth of *A. flavus*, and the number of conidia and hydrophobicity were significantly decreased. Notably, aflatoxin B_1_ biosynthesis and sclerotia production were completely inhibited in the Δ*Afngg1* strain. Furthermore, the pathogenicity of the Δ*Afngg1* strain infecting peanut and corn grains was also diminished, including reduced spore production and aflatoxin biosynthesis compared with *A. flavus* control and *Afngg1* complementation strains. Transcriptome analysis showed that, compared with control strains, differentially expressed genes in Δ*Afngg1* were mainly involved in chromatin remodelling, cell development, secondary metabolism and oxidative stress. These results suggest that Afngg1 is involved in histone 2-hydroxyisobutyrylation and chromatin modification, and thus affects cell development and aflatoxin biosynthesis in *A. flavus*. Our results lay a foundation for in-depth research on the 2-hydroxyisobutyrylation modification in *A. flavus*, and may provide a novel target for aflatoxin contamination prevention.

## 1. Introduction

*Aspergillus flavus*, one of the most abundant and widely distributed fungi on earth, is often found in pre- or post-harvest crops, and it causes serious economic losses [1,2]. It can produce teratogenic and carcinogenic aflatoxins that pose a serious safety hazard to both humans and animals [3]. It is estimated that aflatoxin contamination costs the US corn industry nearly USD 1.68 billion annually [4]. Aflatoxin causes 28% of hepatocellular carcinoma worldwide [5]. Therefore, an in-depth understanding of aflatoxin biosynthetic pathways and their regulatory mechanisms is urgently required to develop effective strategies to control aflatoxin contamination.

Histone post-translational modifications (HPTMs), including acetylation, phosphorylation, ubiquitination and 2-hydroxyisobutyrylation, play a crucial role in many cellular processes in eukaryotes, including secondary metabolite biosynthesis and chromatin regulation [6]. Among them, 2-hydroxyisobutyrylation is a recently discovered post-translational modification that plays an important regulatory role in gene transcription, metabolism and enzyme activity [7]. Previous studies have shown that lysine de-2-hydroxyisobutyrylase CobB can regulate the catalytic activity of enolase by decreasing modification at the K343 site, thereby regulating glycolysis and cell growth in bacteria [8]. In *Ustilaginoidea virens*, mutation of the 2-hydroxyisobutyrylation modification site of mitogen-activated protein kinase UvSlt2 decreased UvSlt2 enzyme activity and significantly reduced the development and virulence of plant pathogenic fungi [9]. Significantly, 2-hydroxyisobutyrylation modification is involved in the pathogenicity of *Botrytis cinerea*, *Candida albicans*, *Fusarium oxysporum* and *Toxoplasma gondii* [7,10,11,12]. Our previous studies have shown that 2-hydroxyisobutyrylation modification regulates mycelial growth and development of *A. flavus*, as well as aflatoxin biosynthesis [13]. These results indicate that lysine 2-hydroxyisobutyrylation modification plays a key role in *A. flavus*, but the enzymes involved in histone 2-hydroxyisobutyrylation modification have not been reported in *A. flavus*. Herein, we identified histone 2-hydroxyisobutyryltransferase Afngg1 in *A. flavus*, and explored its underlying gene regulatory mechanisms using RNA sequencing (RNA-seq)-based transcriptional analysis.

In this study, Afngg1, a homolog of *Candida albicans* histone acetyltransferase Ngg1, was found to be associated with histone 2-hydroxyisobutyrylation modification. Additionally, the functions of Afngg1 in cell development and aflatoxin B_1_ (AFB_1_) production were profiled, and the underlying regulatory mechanisms were investigated through transcriptome analysis. The findings suggest that Afngg1 is a potential target for the effective control of aflatoxin contamination.

## 2. Results

### 2.1. Characterisation of A. Flavus Histone 2-Hydroxyisobutyryltransferase Afngg1

**Figure 1:**
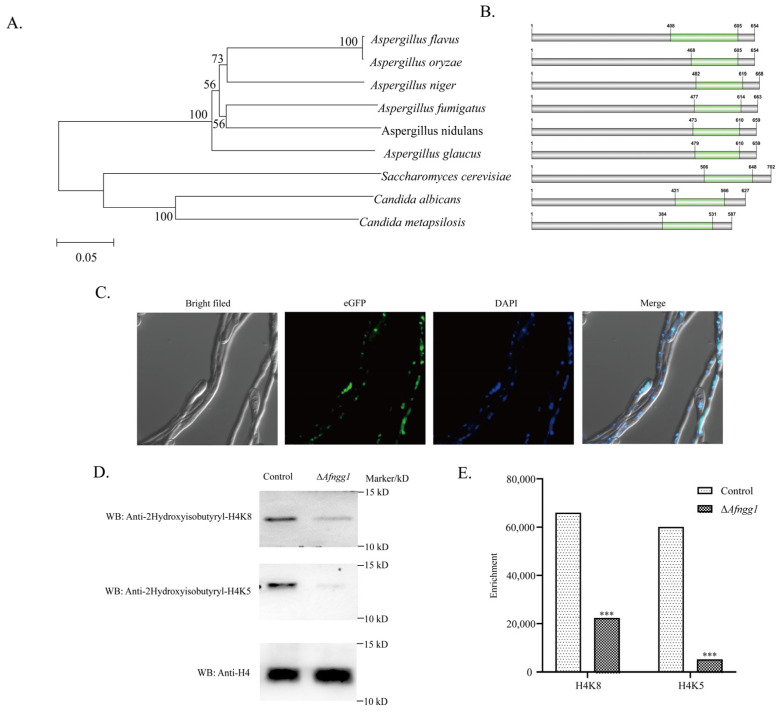
Bioinformatics analysis, subcellular localisation, and modification sites identification of Afngg1. (**A**) Phylogenetic tree based on sequence alignment. The numbers on the branches indicate bootstrap support value. (**B**) The domains of Afngg1 from the above nine species were further visualised by DOG2.0. The green area represented the Ada3 domain. (**C**) Subcellular localization of Afngg1. (**D**, **E**) Level of 2-hydroxyisobutyrylation modification of histones H4K5 and H4K8 in *A. flavus* control and Δ*Afngg1* strains. *** represents *p* < 0.001.

In order to characterise homologs of Afngg1 in *A. flavus*, we obtained homologous sequences by BLAST searching the NCBI database, and constructed phylogenetic trees to analyse their evolutionary relationships. The results revealed that Afngg1 in *A. flavus* is closely related to homologs in other fungi, and most similar to homolog in *A. oryzae* RIB40 (Figure 1A). The analysis also indicated that it was homologous to *Candida albicans* histone acetyltransferase Ngg1. The domains in Afngg1 were further analysed via the NCBI database and visualised using DOG2.0. A conserved Ada3 (histone acetyltransferase subunit 3) domain was found in homologs of Afngg1 proteins in nine species (Figure 1B). Additionally, Afngg1 was localised in the nucleus of *A. flavus* (Figure 1C).

Given the similarity to Ngg1 in *C. albicans*, we speculated that Afngg1 might also be involved in histone modification. To test this hypothesis, the effects of Afngg1 on the level of acetylation of histone H3 and 2-hydroxyisobutyrylation modification of histone H4 were investigated by collecting mycelia of *A. flavus* control and Δ*Afngg1* strains, and proteins were extracted for Western blotting detection using corresponding antibodies. The results indicated that the acetylation modification level of H3K14 in strain ∆*Afngg1* was decreased compared with the *A. flavus* control strain, and notably, acetylation modification of H3K9 was completely absent (Figure S1). Unexpectedly, our results also showed that 2-hydroxyisobutyrylation modification levels of H4K8 and H4K5 were significantly lower than in the *A. flavus* control strain (Figure 1D). These results suggest that Afngg1 contributed to lysine acetylation and 2-hydroxyisobutyrylation as a histone acetyltransferase.

### 2.2. Afngg1 Is Involved in A. flavus Growth, Conidia Formation and Aflatoxin Biosynthesis 

**Figure 2:**
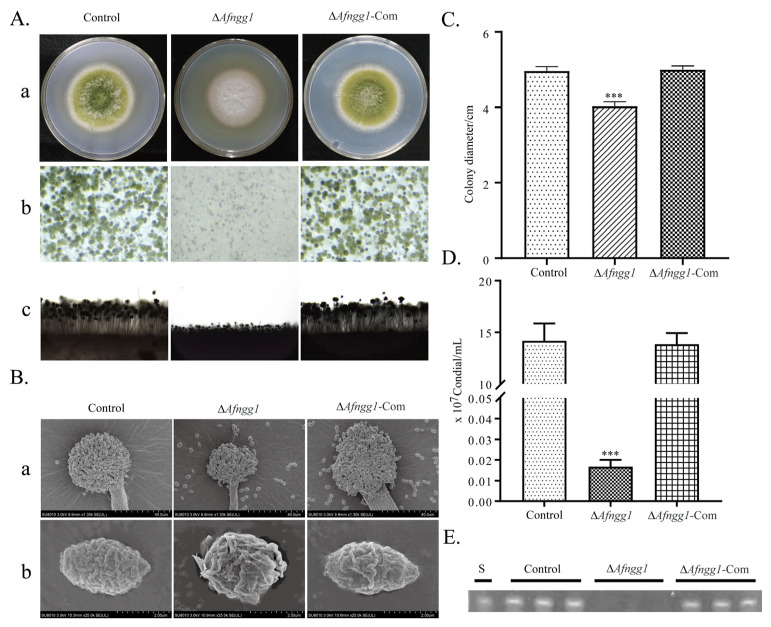
Effects of *Afngg1* on the growth, conidia and aflatoxin biosynthesis of *A. flavus*. (**A**) The colony (**a**), stereoscopic microscope (**b**) and conidiophores (**c**) analysis. *A. flavu*s control strain, Δ*Afngg1* and *Afngg1*-Com strains were cultivated on PDA medium at 37 °C for 5 days. (**B**) SEM analysis of conidia (**a**) and conidial heads (**b**). (**C**) Determination of colony diameter cultured on PDA medium. (**D**) Determination of the number of conidia from different strains. (**E**) TLC analysis of AFB_1_ production. *** represents *p* < 0.001.

To further explore the potential role of *Afngg1* in *A. flavus*, we constructed Δ*Afngg1* and Δ*Afngg1*-Com strains (Figure S2), and the effects of *Afngg1* on *A. flavus* growth, conidia formation and biosynthesis of aflatoxin were evaluated. Unlike the *A. flavus* control strain, mycelia of the ∆*Afngg1* strain were white and fluffy (Figure 2Aa). Stereoscopic microscope observation showed that, compared with the *A. flavus* control strain, conidia heads of the ∆*Afngg1* strain were smaller and fewer in number (Figure 2Ab). Microscopy observation revealed that deletion of *Afngg1* reduced the length of conidiophores and conidial number (Figure 2Ac). SEM results showed that conidial heads of the *A. flavus* control strain were fully formed, while conidial heads of the *Afngg1* strain were significantly smaller and deformed (Figure 2B). Additionally, compared with the *A. flavus* control strain, the mycelia growth rate was decreased after *Afngg1* deletion, and conidia production was decreased by 99.88%, consistent with microscopy observation (Figure 2C,D). Qualitative thin-layer chromatography (TLC) analysis showed that AFB_1_ was no longer produced by the *A. flavus* strain lacking *Afngg1* (Figure 2E).

### 2.3. Effects of Afngg1 on Hydrophobicity and Sclerotia Formation of A. flavus Colonies

**Figure 3:**
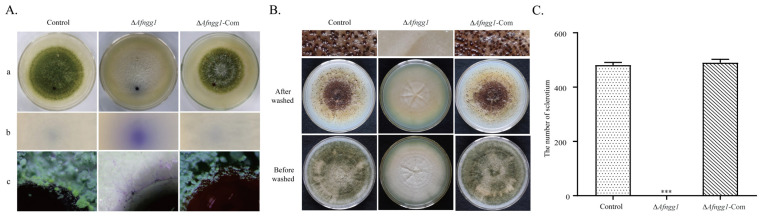
Analysis of hydrophobicity and sclerotium yield. (**A**) Determination of hydrophobicity of *A. flavus* control strain, Δ*Afngg1* and *Afngg1*-Com strains. Colony morphology (**a**), microscope (**b**) and stereoscopic microscopic (**c**) analysis after Bromophenol blue treatment. (**B**) Colony morphology of different strains after 7 days of culture on PDA medium. The plate was sprayed with 70% alcohol to expose the sclerotium. (**C**) Determination of the number of sclerotium in (**B**). *** represent *p* < 0.001.

We studied the effects of *Afngg1* on the surface hydrophobicity of *A. flavus* cells. The results revealed spherical water droplets on *A. flavus* control strain colonies, but these were dispersed and absorbed on Δ*Afngg1* strain colonies (Figure 3(Aa,Ab)). In order to further probe the hydrophobicity differences, the droplet state of mycelia around droplets on colonies and Bromophenol Blue droplets were observed by stereoscopic microscope. The results showed that mycelia and spores around the *A. flavus* control strain were attached to the outer side of the droplet, while Bromophenol Blue solution of the ∆*Afngg1* strain was diffused throughout mycelia (Figure 3(Ac)). Additionally, the results showed that compared with the *A. flavus* control strain, the ∆*Afngg1* mutant did not produce sclerotia at all on PDA medium (Figure 3B,C). These results indicate that *Afngg1* is essential for sclerotia formation in *A. flavus*.

### 2.4. Afngg1 Influences the Infection Ability of A. flavus on Peanut and Corn

**Figure 4:**
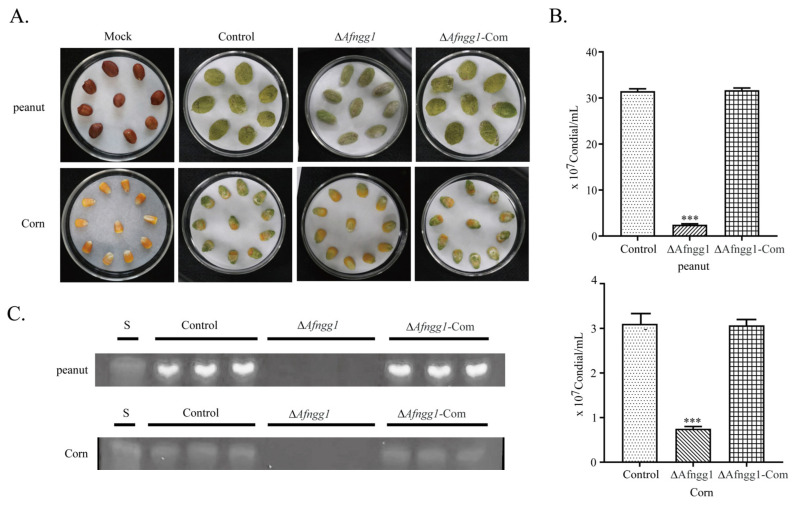
Effect of *Afngg1* on the ability of *A. flavus* to infect crops. (**A**) Colonisation of peanut and corn grains by *A. flavus* control, Δ*Afngg1* and Δ*Afngg1*-Com strains. (**B**) The number of conidia number was determined from the infected peanut and corn grains; (**C**) TLC analysis of AFB_1_ production from infected peanut and corn grains. *** represents *p* < 0.001.

In order to study the pathogenicity of *Afngg1* deletion in *A. flavus*, maize and corn grains were inoculated with spore suspensions. The results showed that the ∆*Afngg1* mutant had a decreased ability to colonise peanut and corn grains, and conidia production was decreased by 97.39% and 91.94%, respectively (Figure 4A,B). We also explored the yield of AFB_1_ on peanut and corn, and AFB_1_ was not detected on either peanut or corn grains infected with the ∆*Afngg1* mutant (Figure 4C). These results indicate that *Afngg1* plays a crucial role in conidia formation of *A. flavus*, and in the biosynthesis of aflatoxin during *A. flavus* colonisation on peanut and corn grains.

### 2.5. Transcriptome Overview

**Figure 5:**
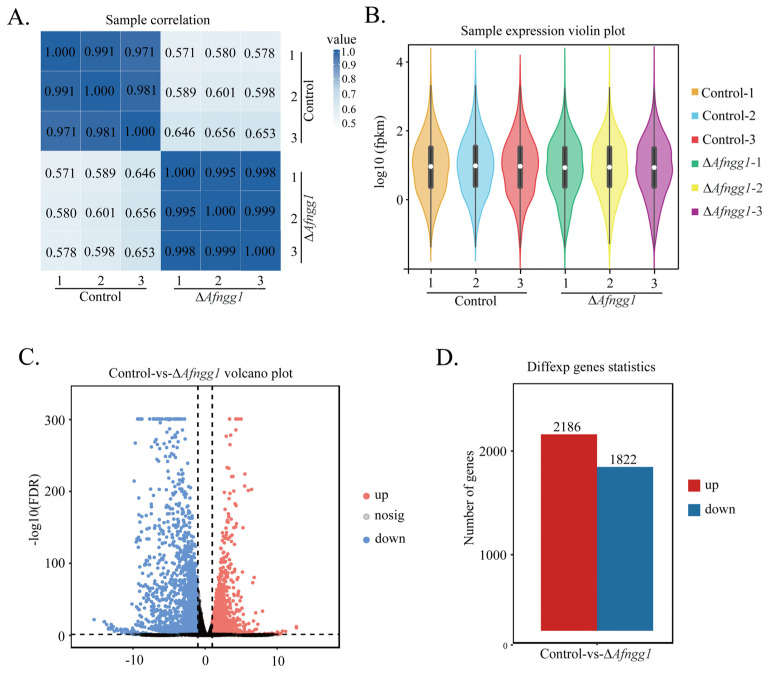
Sample correlation and gene expression analysis. (**A**) Person correlation coefficient. (**B**) Violin plot. (**C**) Volcano map of DEGs. (**D**) Total number of DEGs.

The Pearson correlation coefficient between all pairs of samples was >0.97, suggesting that samples were adequate for subsequent analysis (Figure 5A). Based on the FPKM (Fragments Per Kilobase per Million) value of each gene, gene expression levels were normalised and differentially expressed genes (DEGs) were compared (Figure 5B). The volcano map directly shows the expression ratio and significance of genes (Figure 5C). A total of 4008 significant DEGs were identified, among which 2186 (54.54%) were upregulated and 1822 (45.45%) were downregulated (Figure 5D).

**Figure 6:**
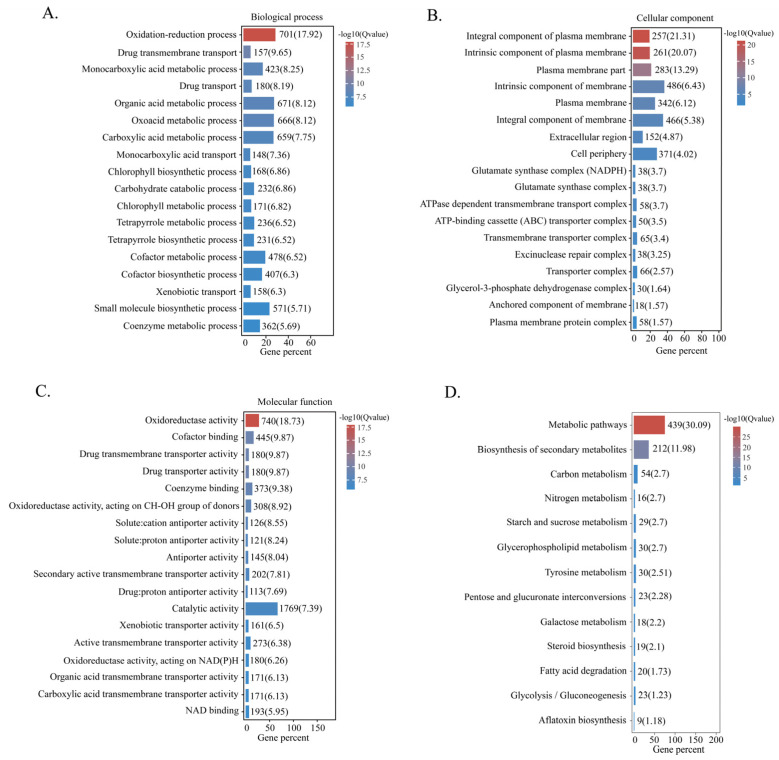
Biological process (**A**), cellular component (**B**), molecular function (**C**) and KEGG pathway (**D**) analysis of DEGs.

The biological functions of DEGs were characterised by GO functional enrichment analysis. The results showed that most DEGs were linked to oxidation–reduction metabolic processes (Figure 6A). In terms of cellular components, DEGs were mainly associated with the plasma membrane, component of membrane and cell periphery subcategories (Figure 6B). The main molecular functions of DEGs were oxidoreductase activity, binding and catalytic activity (Figure 6C). KEGG pathway enrichment analysis results showed that DEGs were mainly involved in metabolic pathways, biosynthesis of secondary metabolites and aflatoxin biosynthesis (Figure 6D). 

### 2.6. Categorisation of DEGs

To further elucidate the regulatory effects of Afngg1 on *A. flavus* cell development and aflatoxin biosynthesis, representative DEGs were divided into four groups: chromatin remodelling, cell development, secondary metabolism and oxidative stress (Table 1). 

Afngg1 deletion altered the expression of genes involved in chromatin remodelling, including histone deacetylases (*clr3*, *RPD3* and *epl1*) and chromatin remodelling complex subunits (*mit1*). Deletion of *Afngg1* inhibited the expression of conidia-related genes *wetA*, *abaA*, *brlA*, *stuA*, *vosA*, *fluG* and *con-6*, and conidia hydrophobicity-related genes *rodA* and *dewA*. Additionally, *ags1*, *ags2*, *MP65*, *chiB1*, *dcw1*, *ecm33*, *erg4, erg7* and *erg5* genes related to cell wall and membrane formation were downregulated.

Secondary metabolism was also affected. The results showed that AFB_1_ biosynthetic pathway genes *aflC*, *aflH*, *aflX*, *aflJ*, *aflW*, *aflQ*, *aflT* and *norA* were downregulated after *afngg1* deletion. Genes associated with the synthesis of other secondary metabolites, including conidia pigments (*pksP* and *pks1*), polyketones (*fluP* and *aurA*), ustiloxin B (*ustYa*, *ustO*, *ustC*, *ustP*, *ustQ*, *ustD* and *ustF2*) and gliotoxin (*gliK*, *gliA* and *gliC*) were also downregulated. Furthermore, *afngg1* deletion inhibited the expression of catalase-related genes *cta1*, *ctl-2* and *cat*.

## 3. Discussion

As an important post-translational modification, 2-hydroxyisobutyrylation plays a vital role in protein synthesis, transcriptional regulation and metabolism [14]. In this study, we performed a functional analysis of histone 2-hydroxyisobutyryltransferase Afngg1 in *A. flavus*. Deletion of *Afngg1* reduced the level of histone 2-hydroxyisobutyrylation modification, indicating that Afngg1 functions as a histone 2-hydroxyisobutyrylyltransferase. Additionally, Afngg1 plays a crucial role in the growth and development of *A. flavus* and the biosynthesis of aflatoxin, and its potential regulatory mechanisms were explored through transcriptome analysis.

### 3.1. Afnng1 Correlates with the Level of Histone 2-Hydroxyisobutyrylation Modification

Previous reports have shown that Ngg1 is required for histone H3 acetylation of *Candida albicans* histone H3, and loss of *Ngg1* greatly reduced the level of acetylation modification of histone H3 in *Ngg1*-A and *Ngg1*-B strains compared with wild-type strains [15]. However, its specific lysine acetylation sites were not characterised. In the present study, we identified homologs of *C. albicans* acetyltransferase NGG1 in *A. flavus*, and identified K9ac and K14ac as the key sites for alteration of histone H3 acetylation. Notably, we also found that Afngg1 is associated with 2-hydroxyisobutyrylation of histone H4, and deletion of *Afngg1* significantly reduced lysine 2-hydroxyisobutyrylation levels at the K5 and K8 sites of histone H4.

### 3.2. Afnng1 Is Involved in Altering Chromatin Remodelling

Histone modification plays a crucial role in altering chromatin structure, among which histone acetylation and 2-hydroxyisobutyrylation modification are important contributors to chromatin remodelling [16,17]. RPD3 is a conserved histone deacetylase that regulates various cellular processes. In *Fusarium graminearum*, deletion of *FgRpd3* led to increased acetylation of histone H4 [18]. Additionally, the *crl3* gene encoding histone deacetylase affects the growth of *Penicillium brasilianum*, and the production of many secondary metabolites [16]. As a chromatin remodelling factor, Mit1 is mainly involved in regulating transcription of heterochromatin regions, and it promotes the deacetylation of histone H3 by clr3 [19]. We found that following deletion of *Afngg1*, expression levels of *clr3*, *RPD3*, *epl1* and *mit1* genes related to chromatin remodelling were altered. Additionally, previous studies have shown that cobB, de-2-hydroxyisobutyrylation enzyme, is involved in regulating the growth and metabolism of prokaryotes [8]. In human cells, deletion of the histone 2-hydroxyisobutyltransferase EP300 decreases the level of 2-hydroxyisobutyrylation modification at specific regulatory sites, and thus reduces the activity of cell metabolism-related enzymes [17]. Our current work showed that histone acetylation modification and 2-hydroxyisobutyrylation levels of the Δ*Afngg1* strain were reduced. These results suggest that Afngg1 may alter chromatin conformation, altering the accessibility of genes, thus affecting cell development and secondary metabolism of *A. flavus*.

### 3.3. Afngg1 Affects Conidial Development and Sclerotium Formation

Recent studies have shown that fungal growth, development and production of secondary metabolites are regulated by post-translational modifications such as 2-hydroxyisobutyrylation [13]. Expression of *A. flavus* conidiogenesis genes is regulated by a cascade involving *brlA*, *abaA* and *wetA* [20]. *BrlA* and *abaA* are key transcription factors, of which *brlA* initiates conidia formation, and *abaA* contributes to conidia development and also affects sclerotia formation and AFB_1_ production [21,22]. *StuA* affects the expression of downstream genes by influencing *brlA* and *abaA*, and regulates sporulation [23]. *VosA*, a member of the velvet protein family, co-regulates spore-specific genes with *wetA*, and is critical for spore maturation and dormancy [24]. Loss of VosA resulted in increased sensitivity to oxidative stress and decreased trehalose content in *A. flavus* conidia [25]. *FluG* plays a balancing role in asexual and sexual development, and deletion of *fluG* can delay and reduce the formation of *A. flavus* conidia [26]; *fluG* can also interact with *velB* or *laeA* to control the production of conidia and sclerotia [27]. In the present study, the genes *wetA*, *abaA*, *brlA*, *stuA*, *vosA*, *fluG* and *con-6* related to conidia were downregulated following deletion of *Afngg1*, mutant strain colonies were white, conidia production was decreased significantly and the strain no longer produced sclerotia. These results suggest that Afngg1 may regulate developmental balance, oxidative stress and conidia trehalose synthesis, and thereby affect the formation of conidia. Additionally, our experiment found that the Δ*Afngg1* strain no longer produced sclerotia, consistent with the results of *fluG* deletion [27]. Transcriptomic analysis also showed downregulation of genes encoding hydrophobic proteins, namely *rodA* and *dewA*, which was confirmed by hydrophobic assay, and is reminiscent of the deletion of laeA in *A. flavus* [28].

### 3.4. Afngg1 Is Required in the Formation of the Cell Wall

The fungal cell wall is a complex structure composed of chitin, dextran and other polymers that play an important role in fungal growth and survival [29]. *Ags1* and *Ags2* are involved in glucan synthesis, and growth was inhibited after *Ags1* deletion [30]. *DCW1* is necessary for incorporation of cell wall glycoproteins into the cell wall, and deletion of the *dcw1* gene in yeast led to cell wall abnormalities [31]. *MP65* encodes β-glucan mannoprotein, which is associated with cell wall integrity and biofilm formation [32]. The GPI-anchored protein Ecm33 is required for cell wall composition, and is involved in fungal cell wall integrity and multiple stress tolerance [33]. One study found that the absence of *ecm33* and *MP65* increased sensitivity to cell wall degraders and altered morphology [32,33]. Herein, transcriptomic results showed that expression levels of the cell wall-related genes *ags1*, *ags2*, *MP65*, *chiB1*, *dcw1* and *ecm33* in the Δ*Afngg1* strain were downregulated. Additionally, ergosterol is an important component of cell membranes, and we found that expression levels of genes associated with ergosterol biosynthesis such as *erg4*, *erg7* and *erg5* were significantly downregulated after deletion of *Afngg1*. These results suggest that Afngg1 may impede the synthesis of cell wall components such as glucan, reduce cell wall tolerance and cause cell wall defects.

### 3.5. Afngg1 Plays a Key Role in Secondary Metabolism

It has been reported that chromatin remodelling is closely related to the biosynthesis of aflatoxin [34]. Aflatoxin biosynthesis includes complex enzymatic reactions that require multiple enzymes, such as fatty acid synthases, polyketone synthases, oxidoreductases, cytochrome P450 monooxygenases, esterases, and others [35]. *AflC* (*pksA*) encodes a polyketone synthase that is involved in the conversion of acetate to norsolorinic acid, and AFB_1_ production can be significantly increased by upregulating *aflC*, *aflR* and *aflK* [36,37]. Both *AflX* and *AflQ* encode oxidoreductases, and *AflQ* is involved in the formation of AFB_1_ precursor hydroxyl-methylsterigmatocystin, and it plays a role in the later stages of the biosynthetic pathway [38,39]. Previous studies revealed a strong linear relationship between *aflQ* expression and aflatoxin biosynthesis [40]. *AflW* encodes a single monooxygenase that catalyses the conversion of hydroxyversicolorone to versiconal hemiacetal acetate [41]. Additionally, *aflS* encodes an esterase (*AflJ*) that interacts with nearby *aflR* genes to regulate the biosynthesis of aflatoxin [42]. Following *aflS* deletion, expression levels of aflatoxin biosynthetic pathway genes including *aflC*, *aflD*, *aflM* and *aflP* were reduced 5–20-fold, and biosynthesis of aflatoxin intermediates was blocked [43]. In the present study, deletion of *Afngg1* downregulated genes encoding enzymes related to the aflatoxin biosynthetic pathway, such as *aflC*, *aflX*, *aflJ*, *aflQ*, *aflT* and *norA*, indicating that Afngg1 can inhibit the biosynthesis of aflatoxin by regulating the expression of multiple genes in the gene cluster via chromatin modification. Our study also found that the Δ*Afngg1* strain no longer produced AFB_1_, consistent with knockout of histone acetyltransferase AflGcnE [6].

Other secondary metabolic pathways are also affected. Ustiloxin B is a secondary metabolite of *Ustilaginoidea virens*, and 18 genes form a gene cluster for the synthesis of ustiloxin B. Deletion of a relevant gene led to a complete or substantial loss of ustiloxin B production in *A. flavus* [44]. Biosynthesis of gliotoxin is carried out by a synthetic gene cluster consisting of 12 genes in *A. fumigatus* [45]. Loss of *gliA* greatly reduced the biosynthesis of glioxin [46], and deletion of *gliK* significantly hindered efflux of glioxins, and increased sensitivity to exogenous glioxins [47]. Herein, we found that expression levels of *ustYa*, *ustO*, *ustC*, *ustP*, *ustQ*, *ustD*, *ustF2*, *gliK*, *gliA* and *gliC*, which are responsible for the biosynthesis of ustiloxin B and gliatoxin, were downregulated. In addition, *A. flavus* produces a variety of polyketone-derived secondary metabolites, which are the most abundant of fungal secondary metabolites [48]. *PksP* was found to be associated with the biosynthesis and virulence of *A. fumigatus* conidia pigments [49]. The *Pks1* gene encodes a polyketone synthase involved in melanin biosynthesis [50]. Deletion of polyketone synthase gene *Flup* resulted in decreased filament growth rate and conidial production in *A. parasitica* [51]. The present study found that expression levels of polyketone synthase-related genes *fluP*, *pksP*, *aurA* and *Pks1* were downregulated. These results suggest that Afngg1 may play an important regulatory role in mycotoxin efflux and self-protection.

### 3.6. Afngg1 Alters Oxidative Stress Homeostasis

Oxidative stress is often associated with the synthesis of fungal secondary metabolites. Biosynthesis of aflatoxin has been reported to be inhibited by increased reactive oxygen species (ROS) levels and decreased activity of antioxidant enzymes [52]. Cells produce a variety of antioxidant enzymes to remove excessive ROS to protect cells from oxidative stress [53]. Previous reports have shown that deletion of *cat1* significantly increased intracellular ROS levels, which in turn increased oxidative stress levels and reduced aflatoxin biosynthesis and virulence [52]. In our current work, expression levels of catalase-related genes *cat1*, *ctl-2* and *cat* were downregulated, suggesting that deletion of *Afngg1* might prevent cells from clearing ROS in a timely manner, thereby diminishing their defences against oxidative stress, and inhibiting the biosynthesis of aflatoxin.

In conclusion, we identified histone 2-hydroxyisobutyryltransferase Afngg1 in *A. flavus*, and explored its potential roles in cell development and aflatoxin biosynthesis. The results indicated that deletion of *Afngg1* reduced the degree of lysine 2-hydroxyisobutyrylation modification of histone H4, inhibited cell growth, conidia formation, sclerotia production and AFB_1_ production in *A. flavus*, and decreased hydrophobicity. RNA-seq was subsequently used to investigate the underlying regulatory mechanisms, and analysis of DEGs showed that genes associated with chromatin remodelling, cell development, oxygen stress and biosynthesis of aflatoxin, ustiloxin B and gliatoxin, were downregulated. Therefore, these results suggest that histone 2-hydroxyisobutyryltransferase Afngg1 affects chromosomal modification, alters gene expression and regulates the growth, development and aflatoxin biosynthesis of *A. flavus*. The findings enhance our understanding of the role of histone 2-hydroxyisobutyrylation in aflatoxin biosynthesis and fungal pathogenicity regulation, and provide potential targets for exploring new control strategies for *A. flavus*.

## 4. Materials and Methods

### 4.1. Strains and Culture Conditions

*A. flavus* CA14 (*kusA^−^*, *pyrG^+^*) served as a control strain, and the *Afngg1* knockout strain (Δ*Afngg1*), *Afngg1* complementation strain (Δ*Afngg1*-Com) and Afngg1-enhanced green fluorescence protein (eGFP) strain were derived in this study. Potato dextrose agar (PDA) was used to evaluate growth, conidia formation, cell hydrophobicity and aflatoxin B_1_ production, with appropriate amounts of uridine (10 mmol/L) added to the medium as needed. For sclerotia production, spore suspensions were maintained on PDA medium at 37 °C for 7 days in the dark. Each experiment was repeated three times.

### 4.2. Sequence Resources and Phylogenetic Tree Analysis

We used the protein sequence of Afngg1 to perform a BLAST search to identify homologous sequences in the National Center for Biotechnology Information (NCBI) database. Afngg1 domains were predicted by DOG 1.0 software (Ren and Wen, Hefei, China). Afngg1 protein sequences were aligned by ClustalW using MEGA software (7.0 version, iGEM, Temple University, Philadelphia, PA, USA), and a neighbour-joining phylogenetic tree was constructed [54]. 

### 4.3. Construction of ΔAfngg1, ΔAfngg1-Com and Afngg1-eGFP Strains

The Δ*Afngg1* strains were constructed using homologous recombination with PCR primers listed in Table S1. PCR amplification was performed using P505 DNA polymerase (Vazyme, Nanjing, China). For construction of the Δ*Afngg1* strain, primers Δ*Afngg1*-1F, Δ*Afngg1*-1R, Δ*Afngg1*-2F and Δ*Afngg1*-2R were used to amplify the 1.1 kb upstream and 1.5 kb downstream regions of *Afngg1* fragments in the *A. flavus* genome, respectively. Meanwhile, primers pyrG-F and pyrG-R were used to amplify the pyrG selection marker. Fusion PCR was performed using primers Δ*Afngg1*-1F and Δ*Afngg1*-2R to generate a fragment containing the upstream and downstream segments, and the pyrG selection marker [55]. The fusion fragment was purified and transformed into the *A. flavus* control strain according to previous methods [34], and transformants were selected for verification.

Construction of the *Afngg1*-Com strain was performed according to previous methods [56]. Primers Δ*Afngg1*-Com-F and Δ*Afngg1*-Com-R were used to amplify a fragment including the promoter, coding sequence and terminator from *A. flavus* control strain genomic DNA, and this was ligated to the pPTRI plasmid following *Hind*III and *Sma*I restriction enzyme digestion. The resulting plasmid was transferred into *A. flavus* Δ*Afngg1* protoplasts, and the Δ*Afngg1*-Com strain was confirmed by PCR. 

For construction of the Afngg1-eGFP strain, linker, eGFP and TglaA were sequentially connected next to the Afngg1 coding sequence without a stop codon, using primers Afngg1-eGFP-2F and Afngg1-eGFP-2R. The fragment was ligated to the pPTRI plasmid according to previous methods [52]. The confirmed plasmid was transferred into Δ*Afngg1* protoplasts, and transformants were selected for verification.

### 4.4. Subcellular Localisation

To detect subcellular localisation of Afngg1, *A. flavus* spore suspensions (10^6^) were inoculated into Dextrose Peptone Yeast (DPY) liquid medium and cultured at 30 °C for 24 h. After collection, mycelia were washed with phosphate-buffered saline (PBS), stained with 4,6-diamidino-2-phenylindole for 10 min according to a previous method [57], and observed with an FV1000 laser confocal microscope (Olympus, Beijing, China).

### 4.5. Protein Extraction and Western Blotting Analysis

To explore the effect of *Afngg1* deletion on the acetylation level of histone H3 and the 2-hydroxyisobutyrylation level of histone H4 in *A. flavus*, *A. flavus* control and Δ*Afngg1* strains were cultured on PDA medium at 30 °C for 72 h. Mycelia were frozen with liquid nitrogen and ground to a powder. An appropriate amount of each protein sample was mixed with sample buffer and protein lysis solution to make a final concentration of 1–2 mg/mL, and heated at 95 °C for 10 min prior to sodium dodecyl sulphate polyacrylamide gel electrophoresis (SDS-PAGE). Proteins were transferred to a PVDF membrane, and this was blocked with 5% skimmed milk powder after transfer. For analysis of lysine acetylation of histone H3, anti-H3K9ac, anti-H3K14ac and anti-H3 were used as primary antibodies. For analysis of lysine 2-hydroxyisobutyrylation, anti-H4K5_hib_ (PTM-854; 1:10,000 dilution; PTM-Bio, Hangzhoou, China) and anti-H4K8_hib_ (PTM-805; 1:10,000 dilution; PTM-Bio, Hangzhoou, China) were used as primary antibodies. Goat anti-mouse IgGI (1:10,000 dilution; Thermo, Shanghai, China) was used as secondary antibody, and chemiluminescent horseradish peroxidase (HRP) substrate (Millipore) was added and incubated for 2 min for signal detection. Meanwhile, protein gray scale analysis was performed using Image-Pro Plus software (4.5 version, Media Cybernetics, Silver Spring, USA) according to a previous method [6].

### 4.6. Morphological and Physiological Analyses

To investigate the effect of *Afngg1* on the growth of *A. flavus*, 2 μL spore suspensions (10^6^ spores/mL) of *A. flavus* control, Δ*Afngg1* and Δ*Afngg1*-Com strains were separately inoculated in the centre of PDA plates and cultured at 30 °C for 5 days; then, the colony diameter was measured and conidial heads were counted under a stereoscopic microscope (Nikon, Shanghai, China). Next, 6 mL sterile water was added to each Petri dish to harvest the mycelium, and the number of conidia was calculated using a hemocytometer. To assess the hydrophobicity of *A. flavus* mycelia, 20 μL sterile water and 20 μL 3% Bromophenol Blue solution were added to the edges of colonies of *A. flavus* control, Δ*Afngg1* and Δ*Afngg1*-Com strains, cells were cultured at 30 °C for 2 days, and analysed by a stereoscopic microscope. Quantitative analysis of sclerotia production was performed at 37 °C for 7 days. After 7 days, 75% ethanol was sprayed to wash the medium and expose the sclerotia, and five holes were made along the diameter to count the number of sclerotia. Additionally, scanning electron microscopy (SEM) was used to photograph the conidia microstructure of *A. flavus* control, Δ*Afngg1* and Δ*Afngg1*-Com strains as described previously [58].

### 4.7. Determination of AFB_1_ Production

AFB_1_ production analysis was carried out according to a previously described method [59]. Spore suspensions (10^6^ spores/mL) of *A. flavus* control, Δ*Afngg1* and Δ*Afngg1*-Com strains were cultured in 50 mL Yeast Extract Sucrose (YES) liquid medium at 30 °C in the dark for 5 days. An equal volume of chloroform was added to extract aflatoxin from the 50 mL medium. After the chloroform was completely volatilised, the residue was dissolved in 1 mL methanol and then analysed by TLC. The AFB_1_ yield of *A. flavus* strains was evaluated using an AFB_1_ standard sample (0.1 mg/mL).

### 4.8. Peanut and Corn Infection Assay

A peanut and corn infection experiment was performed as described previously [60]. Peanut and corn seeds were inoculated with 10^6^/mL spore suspensions of *A. flavus* control, Δ*Afngg1* strain and Δ*Afngg1*-Com strain and cultured at 28 °C in the dark for 6 days, with water replenished each day. Sterile water was added to collect the spore suspension and the number of conidia was calculated using a hemocytometer according to a previous method [34]. Finally, chloroform was added to extract aflatoxin as described above.

### 4.9. Transcriptome Analysis

Total RNA was extracted using Trizol reagent (Thermo Fisher, Shanghai, China) following the manufacturer’s instructions. RNA quality and integrity analyses, cDNA library construction, and RNA-Seq were performed by Guangzhou Gene Denovo Biotechnology (Guangzhou, China). The Pearson correlation coefficient was used to analyse the accuracy and reliability of the three biological duplicate samples. DESeq was used to analyse differences in FPKM value, and differences were considered statistically when log2(fold change) >1 and *p*-value < 0.05 criteria were met. Genome Ontology (GO) functional analysis and Kyoto Encyclopedia of Genes and Genomes (KEGG) pathway analysis were carried out using FungiFun (https://sbi.hki-jena.de/FungiFun/FungiFun.cgi) (accessed on 15 August 2022) and the KEGG Automatic Annotation Sever (http://www.kegg.jp/ or http://www.genome.jp/kegg/) (accessed on 15 August 2022).

## Figures and Tables

**Table 1:** Classification of representative DEGs in the Δ*Afngg1* strain vs. control strain.

Gene Category	Name	Log2(fc)	*p*-Value	Description
Chromatin remodelling				
AFLA_025120	clr3	1.089	2.82 × 10^−21^	histone deacetylase
MSTRG.3596	RPD3	1.15	1.26 × 10^−6^	histone deacetylase RPD3
AFLA_055490	epl1	1.51	5.53 × 10^−44^	histone acetyltransferase complex component
AFLA_052300	mit1	1.49	7.93 × 10^−28^	chromatin remodelling complex subunit (Chd3)
Cell development				
AFLA_052030	wetA	−2.32	1.96 × 10^−120^	developmental regulatory protein WetA
AFLA_029620	abaA	−3.17	1.99 × 10^−171^	transcription factor abaA
AFLA_082850	brlA	−5.27	0	C2H2 type conidiation transcription factor
AFLA_046990	stuA	−1.03	5.33 × 10^−46^	APSES transcription factor StuA
AFLA_026900	vosA	−2.28	1.98 × 10^−75^	developmental regulator VosA
AFLA_039530	fluG	−3.17	1.28 × 10^−13^	FluG family protein
AFLA_044800	con-6	−8.10	7.93 × 10^−60^	conidiation protein Con-6
AFLA_098380	rodA	−7.58	2.79 × 10^−67^	RodA/RolA7
AFLA_060780	dewA	−6.81	8.80 × 10^−9^	hydrophobin family protein
AFLA_023460	ags1	−2.80	6.03 × 10^−201^	alpha-1,3-glucan synthase Ags1
AFLA_134100	ags2	−2.28	4.34 × 10^−119^	alpha-1,3-glucan synthase Ags2
AFLA_052780	MP65	−3.87	1.26 × 10^−141^	cell wall glucanase (Scw4)
AFLA_104680	chiB1	−1.64	1.57 × 10^−22^	class V chitinase ChiB1
AFLA_024280	dcw1	−5.49	0	cell wall glycosyl hydrolase Dfg5
AFLA_113120	ecm33	−1.02	6.90 × 10^−76^	GPI-anchored cell wall organization protein
AFLA_138060	erg4	−2.95	1.32 × 10^−108^	c-24(28) sterol reductase
AFLA_001030	erg7	−9.21	1.07 × 10^−73^	lanosterol synthase
AFLA_028640	erg5	−5.79	2.08 × 10^−143^	cytochrome P450 sterol C-22 desaturase
Secondary metabolism				
AFLA_139410	aflC	−1.00	3.76 × 10^−32^	aflC/pksA/pksL1/polyketide synthase
AFLA_139330	aflH	−1.46	2.3 × 10^−3^	aflH/short chain alcohol dehydrogenase
AFLA_139160	aflX	−2.37	1.05 × 10^−2^	aflX/ordB/monooxygenase/oxidase
AFLA_139320	aflJ	−2.82	1.28 × 10^−2^	aflJ/estA/esterase
AFLA_139170	aflW	−3.82	6.16 × 10^−6^	aflW/moxY/monooxygenase
AFLA_002920	aflQ	−4.82	2.29 × 10^−46^	flavonoid 3-hydroxylase
AFLA_024090	aflT	1.57	8.06 × 10^−24^	efflux pump antibiotic resistance protein
AFLA_139310	norA	−8.34	7 × 10^−4^	aflE/norA/aad/adh-2/ NOR reductase
AFLA_094990	ustYa	−7.92	2.80 × 10^−21^	Ustiloxin B biosynthesis protein Ya
AFLA_094940	ustO	−2.98	2.18 × 10^−108^	Ustiloxin B biosynthesis protein O
AFLA_094960	ustC	−4.14	1.27 × 10^−48^	cytochrome P450
AFLA_095010	ustP	−4.59	1.18 × 10^−84^	Ustiloxin B biosynthesis protein P
AFLA_095060	ustQ	−4.75	1.78 × 10^−35^	tyrosinase
AFLA_095040	ustD	−6.18	9.10 × 10^−2^	NRPS-like enzyme
AFLA_095050	ustF2	−7.29	5.02 × 10^−20^	dimethylaniline monooxygenase
AFLA_064420	gliK	−3.26	3.49 × 10^−12^	gliotoxin biosynthesis protein GliK
AFLA_118990	gliA	−4.91	1.47 × 10^−127^	efflux pump antibiotic resistance protein
AFLA_064540	gliC	−6.69	1.62 × 10^−55^	cytochrome P450 oxidoreductase
AFLA_114820	fluP	−1.44	1.78 × 10^−50^	polyketide synthase
AFLA_006170	pksP	−6.54	0	polyketide synthetase PksP
AFLA_010000	aurA	−2.23	2.02 × 10^−84^	polyketide synthase
AFLA_054090	pks1	−2.22	7.5 × 10^−3^	polyketide synthase
Oxidative stress				
AFLA_034380	cta1	−7.68	0	catalase
AFLA_096210	ctl-2	−6.96	6.43 × 10^−272^	catalase
AFLA_100250	cat	−1.57	6.97 × 10^−16^	catalase

Means with *p*-value < 0.05 are considered to have significant difference.

## Data Availability

The raw transcriptome read data are available in the SRA database under accession number SUB12287216.

## Supplementary Materials

The following supporting information can be downloaded at: https://www.mdpi.com/article/10.3390/toxins15010007/s1, Figure S1: Determination of acetylation modification levels of histone H3K9ac and H3K14ac in *A. flavus* control and Δ*Afngg1* strains; Figure S2: Construction and verification of Δ*Afngg1* strain, complement and localisation strains; Table S1: PCR primers were used in this study.
