# Supplementary material for: Histone 2-Hydroxyisobutyryltransferase Encoded by Afngg1 Is Involved in Pathogenicity and Aflatoxin Biosynthesis in Aspergillus flavus
Source: Toxins (Basel). 2022 Dec 21;15(1):7. doi: 10.3390/toxins15010007 (PMC9861817; doi:10.3390/toxins15010007)
Supplement: Supplementary file 1 [file toxins-15-00007-s001.zip › toxins-2083452-supplementary.pdf]

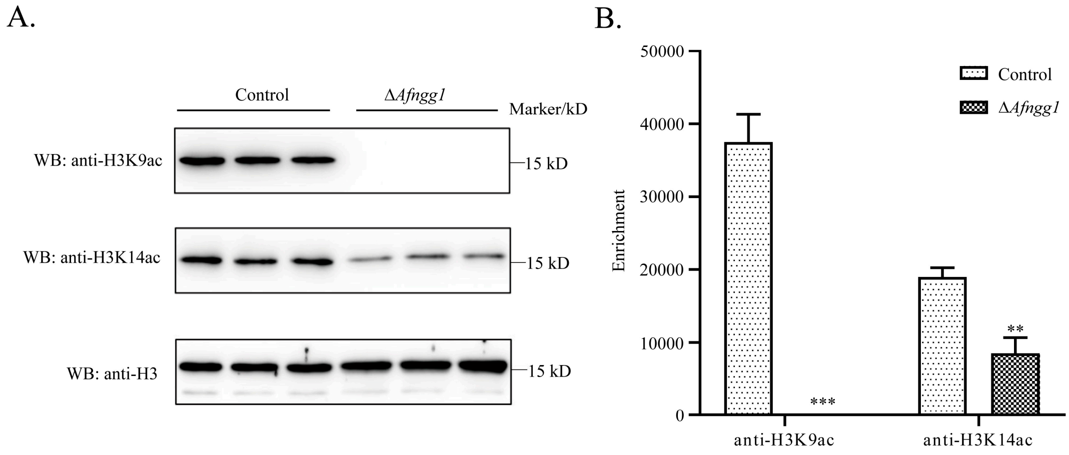

Figure S1. Determination of acetylation modification levels of histone H3K9ac and H3K14ac in *A. flavus* control and Δ*Afngg1* strains. (**A**) The acetylation levels of H3K9 and H3K14 were determined using anti-acetyl H3K9 and anti-acetyl H3K14 (ac-H3K14) antibody with anti-acetyl histone 3 as a control. (**B**) Enrichment levels of (A) by the bank optical density method. ** and *** represented $p < 0.05$ and $p < 0.001$, respectively.

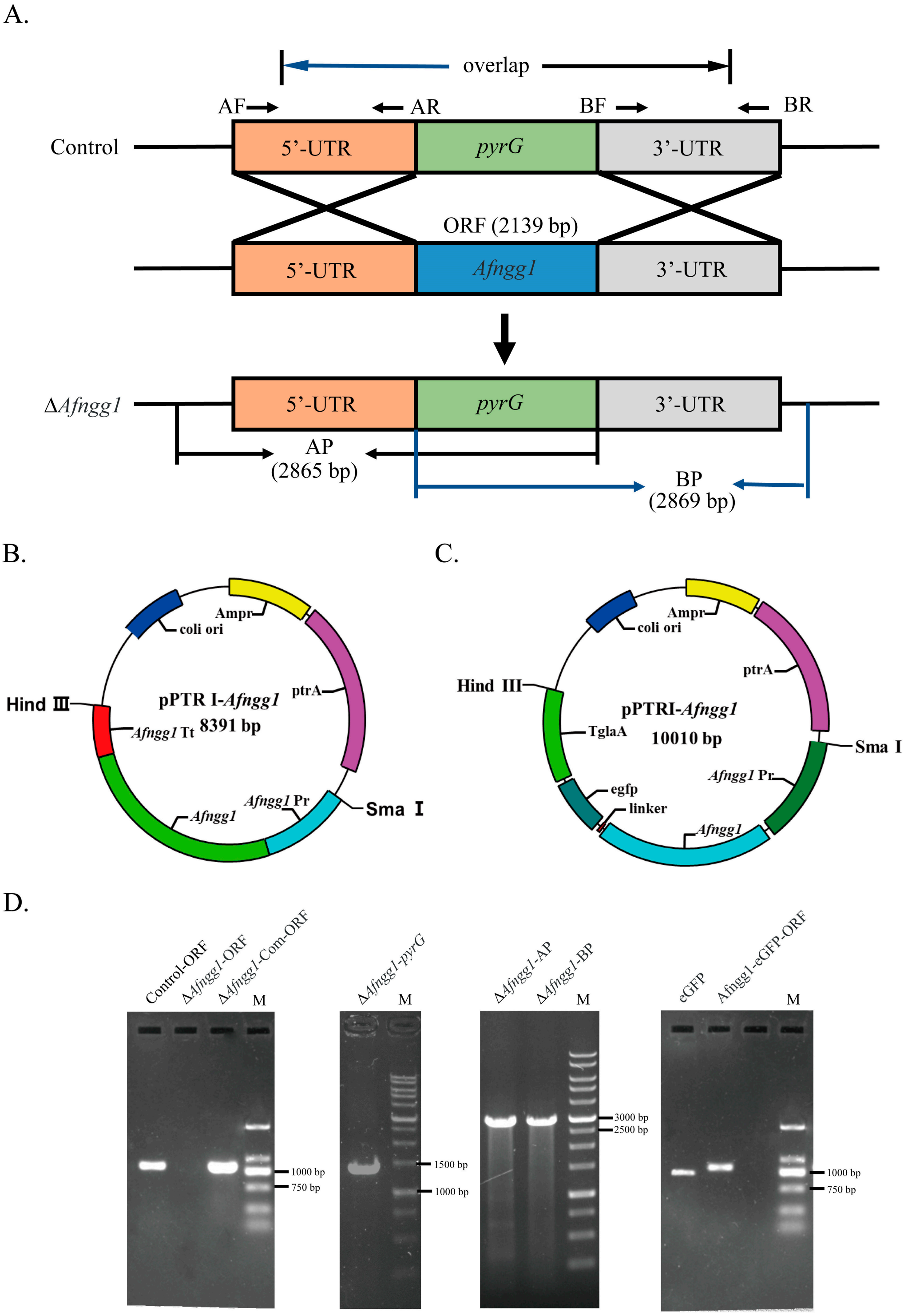

Figure S2. Construction and verification of Δ*Afngg1* strain, complement and localization strains. (**A**) Schematic diagram of Δ*Afngg1* strain construction. (**B**) Plasmid for *Afngg1* complementation. (**C**) Plasmid for *Afngg1* localization. (**D**) Verification of *Afngg1* deletion, complementation and localization strains.

Table S1. PCR primers were used in this study.

Name	Sequence (5’-3’)
Δ*Afngg1*-1F	ctagaggatctactagtcatatggattATGAATGGAGCGGTGAGATTGTTGC
Δ*Afngg1*-1R	ctgcggcgcgttctcgaggaagttgcTGTGATGACGGCCCGAAAAGGTGTT
Δ*Afngg1*-2F	atgtgtaacggtattgactaaaagggTTCTTCGCTTGCTCCTCATGCAGTTTT
Δ*Afngg1*-2R	tcgagctcggtacccggggatccgattGCACGTCCCCTTGTTCACTGTTCGATA
Δ*Afngg1*-iden-F	GCGAGGCAGATGATGAAGAGGAGGAAT
Δ*Afngg1*-iden-R	AGAATACAGGCGGCTAACTAATGGACC
Δ*Afngg1*-Com-F	AAACAGCTATGACCATGATTACGCCATGCGCGGTCGAGACAGAATCTAAAG
Δ*Afngg1*-Com-R	GGCCAGTGAATTCGAGCTCGGTACCCAGGTAACCAGCGGTCCATCATCCAG
Afngg1-eGFP-1F	AAACAGCTATGACCATGATTACGCCAGCAGCAGCAGGTGGAGACATTATTG
Afngg1-eGFP-1R	CTCCTCATCCCAACCTTCAACTTCC
Afngg1-eGFP-2F	GGAAGTTGAAGGTTGGGATGAGGAGGGCGCAGGCGCCGGCGCAGGCGCCGGC
Afngg1-eGFP-2R	GGCCAGTGAATTCGAGCTCGGTACCCCCGGAGATCCTGATCATCCGTGAGAA
pyrG-F	GCCTCAAACAATGCTCTTCACCC
pyrG-R	GTCTGAGAGGAGGCACTGATGC
